# Sanitary

**Published:** 1882-01

**Authors:** 


### SANITARY.

In the fourteenth annual report of the Health Department of Cincinnati, the following facts are presented, viz.: Of the deaths occurring during the year 1880, 720 were from consumption, 697 from pneumonia and other lung diseases; 430 diarrhœal diseases; 178 typhoid fever; 134 scarlet fever; 103 diptheria; 103 measles. 1,281 were from zymotic diseases. Of the total number from all causes 2,192, or 42.35 per cent., were of children under five years of age.

What a fearful slaughter of the innocents. From health reports it is quite apparent that about one half of the race die in infancy, or before five years of age. The question arises, why is this so? In answer to this the conviction will force itself upon every close observer, that it is more from carelessness and neglect than from ignorance. When will the public wake up to the importance and magnitude of sanitary science?

